# Effects of Nickel at Environmentally Relevant Concentrations on Human Corneal Epithelial Cells: Oxidative Damage and Cellular Apoptosis

**DOI:** 10.3390/biom12091283

**Published:** 2022-09-12

**Authors:** Zhen-Ning Zhang, Hai Liu, Mi-Mi Liu, Dan-Lei Yang, Jue Bi, Qian-Qian Chen, Wei Chen, Ping Xiang

**Affiliations:** 1 Yunnan Province Innovative Research Team of Environmental Pollution, Food Safety, and Human Health, Institute of Environmental Remediation and Human Health, School of Ecology and Environment, Southwest Forestry University, Kunming 650224, China; 2 Affiliated Hospital of Yunnan University, Eye Hospital of Yunnan Province, Kunming 650224, China; 3 The Affiliated Ningbo Eye Hospital, Wenzhou Medical University, Ningbo 315040, China

**Keywords:** nickel, human corneal epithelial cells, oxidative stress, apoptosis, ocular damage

## Abstract

Nickel (Ni) is ubiquitous in the environment and evidence has suggested that Ni can cause ocular surface inflammation, especially in fine particulate matter and personal products. Continuous daily exposure to Ni-containing dust may adversely impact the human cornea, whereas the underlying mechanism of this phenomenon remains not fully understood. Here, human corneal epithelial cells (HCEC) were employed to analyze the toxicity of Ni via detections of cell morphology, cell viability, reactive oxygen species production, cell apoptosis rate, and apoptotic gene expression levels after exposure for 24 h to uncover the damage of Ni to the cornea. A concentration-dependent inhibition of HCECs’ viability and growth was observed. In particular, Ni at 100 μM significantly decreased cell viability to 76%, and many cells displayed an abnormal shape and even induced oxidative damage of HCEC by increasing ROS to 1.2 times, and further led to higher apoptosis (24%), evidenced by up-regulation of apoptotic genes *Caspase-8*, *Caspase-9*, *NF-κB*, IL-1β, and *Caspase-3*, posing a risk of dry eye. Our study suggested that Ni induces apoptosis of HCEC through oxidative damage. Therefore, Ni pollution should be comprehensively considered in health risks or toxic effects on the ocular surface.

## 1. Introduction

Nickel (Ni) is a possibly essential element for the human body and has the role of maintaining physiological structure, stimulating blood production, and affecting enzyme activity [1], but its demand is exceedingly small, once overstepping the limit will endanger human health. Ineluctably, as a trace metal, Ni has been widely detected in numerous environmental media in the form of different compounds due to its high ductility and corrosion resistance, such as personal products, water, soil, dust, and fine particulate matter [2,3,4]. Even in the water extract of indoor dust, Ni concentration can reach 89.2 mg/kg [5].

Moreover, studies have proven that Ni can pose potential harm to human health, such as immunotoxicity, neurotoxicity, teratogenicity, developmental toxicity, and reproductive toxicity [3,6,7]. More frightening is that epidemiological reports have proved that occupational Ni exposure could lead to a high incidence of nasal and lung cancers [8,9]. As early as 1990, Ni and its compounds were classified as first-class carcinogens to humans by WHO’s International Agency for Research on Cancer (IARC) [10]. Interestingly, Ni is the essential element for most organisms as well, so it is impossible to avoid its widespread appearance in modern industries such as refining, electroplating, welding, and the production of nickel-cadmium batteries, or even in food people live on such as vegetables, nuts, and chocolate [3,11]. In consideration of oxidate and corrosion resistance, Ni is rather difficult to degrade, which poses an extremely high health risk.

What is worth mentioning is that Ni is frequently detected in personal products such as cosmetics and eyeglasses, for example, Ni can be added as the component of preservatives, dyestuffs, and fragrances to eye shadow and eyeliner, in which the amount is greater than the recommended amount of 1 ppm (16.95 μM), with the prevalence of eyelid dermatitis ranging from 30 to 77% [3,4], suggesting it is a potential threat to our ocular surface for its common contact with the eyeball.

Beyond that, insoluble particles in the air are also the common carriers for this metal. Ni can exist as the components of dust and smoke, and both carry a risk of corneal injury [12,13]. As early as 1997, nickel has been shown as one of the most abundant heavy metals in residential oil fly ash [14] and even accounts for over 30% of the total amount of heavy metals [15]. According to previous studies, the concentration of Ni in indoor dust varied from 1.09 to 102.20 μg/g [16,17], and our team has confirmed that water extract containing a relatively large amount of Ni had adverse impacts on human eyes [5].

The ocular surface, as the first barrier between the eyeball and the outside world, will be directly exposed to the pollutants in air, dust, water, and other carriers. The ocular surface consists of the tear film, the epithelia of the cornea, and conjunctiva, while the cornea is the most anterior tissue of the eye surface [18], once exposed to pollutants, it will cause inflammation or even more serious lesions such as dry eye [19]. Since the 1970s, Ni damage to the ocular surface has been reported [20], but subsequent studies on Ni and the ocular surface have not been conducted, and few have been done on the cornea.

A report indicated that cells generally uptake Ni as divalent ions [1]. In deeper mechanisms at the cellular and molecular level, Ni exposure relates to the production of reactive oxygen species in human cells [2], resulting in oxidative damage. Eventually, it can cause apoptosis [21,22,23]. Thus, evaluating the negative effect of Ni on human corneal epithelial cells (HCEC) is of great importance. Naveen Puttaswamy and Karsten Liber investigated the toxic effects of nickel salts with different anions (HCO_3_^−^, Cl^−^, and SO_4_^2−^). Experiments showed that SO_4_^2−^ enhanced the activity of metals, HCO_3_^−^ accelerated the release of oxidized metals, while Cl^−^ has no special effect on the type and amount of metals released, which can reflect the effect of exposure more truthfully [24]. So, we measured cell viability, intracellular ROS levels, cell apoptosis rate, and apoptotic gene expression levels of HCEC after being exposed to NiCl**_2_** for 24 h.

## 2. Materials and Methods

### 2.1. Chemicals and Reagents

Dulbecco’s Modified Eagle Media: Nutrient Mix F-12 (DMEM/F-12), Fetal Bovine Serum (FBS), and Phosphate-Buffered Saline (PBS) were from Procell Life Science & Technology Co., Ltd. (Wuhan, China); antibiotic-antimycotic solution and epidermal growth factor (EGF) were from Gibco and Life Technologies (Thermo Fisher, Waltham, MA, USA), respectively. Cell culture plates including 96-well and 6-well plates were all from Corning Inc. (New York, NY, USA). The CCK-8 cell viability assay kit was from GlpBio Technology, Ltd. (Montclair, NJ, USA). Trypsin-EDTA Solution (containing 0.25% Trypsin) was from Biosharp Life Sciences (Hefei, China). The Reactive Oxygen Species Assay Kit was from Beyotime Biotechnology Co., Ltd. (Shanghai, China). Cell apoptosis detection kits, total RNA extraction reagent, Script 1st Strand cDNA Synthesis Kit (gDNA Eraser), and SYBR green qPCR master mix were all from Yi Fei Xue Biotech, Ltd. (Nanjing, China). Nickel chloride (NiCl_2_·6H_2_O) reference was bought from Rhawn.

### 2.2. Cell Culture and Cell Exposure

Human corneal epithelial cells (HCEC) were from the same donor and given by Dr. Zheng from the Eye Hospital of Wenzhou Medical University. Cells at passage 18 were cultured in Dulbecco’s Modified Eagle Media: Nutrient Mix F-12 supplemented with 10% FBS, 1% antibiotic-antimycotic solution, and EGF (10 ng/mL) in the constant temperature incubator containing 5% CO_2_ at 37 °C (Thermo Fisher 371, USA). After cell passage, the morphology of HCEC was observed to be normal by microscope and 80% of the plate was covered, then different concentrations of HCEC were inoculated into a 6-well plate (1 × 10^6^ cells/100 μL/well) and 96-well plate (1 × 10^4^ cells/2 mL/well) according to the requirements for different purposes for exposure.

After a preliminary experiment to ensure the lethal concentration, we chose 1 μM, 10 μM, and 100 μM as exposure concentrations of NiCl_2_. HCEC were placed into a 96-well plate (100 uL/well) at a density of 1 × 10^4^ cells/mL and incubated at 37 °C for 24 h, then were exposed to NiCl_2_ after being washed with PBS. We dissolved NiCl_2_ in ultrapure water to prepare a stock solution of 100 mM. After being sterilized by a 0.22 μm filter, the solution was diluted with DMEM/F-12 to the set concentrations. Ni^2+^ liquids of different concentrations were added to the plates and incubated for 24 h.

### 2.3. Cell Morphology and Viability Assay

Morphological changes in cells were observed and photographed by an inverted microscope (TS-100, Nikon, Japan) at 200× magnification after exposure to Ni in the 96-well plate, and then the CCK-8 assay kit was employed to detect cell viability according to the manufacturer’s instructions. We added 10 μL CCK-8 solution to each well in the plate in case of introducing bubbles and incubated the plate for another 2 h. The absorbance was measured with a microplate reader (SpectraMax^®^ Plus 384, Molecular Devices, San Jose, CA, USA) at 450 nm.

### 2.4. Measurement of ROS

The Reactive Oxygen Species Assay Kit using fluorescent probe DCFH-DA was applied to detect the level of intracellular reactive oxygen species. Based on the instructions, after 24 h exposure, we washed the HCEC in the 6-well plate with PBS twice to remove the exposure fluid, then treated cells with 1 mL fluorescent probe DCFH-DA at 10 μM each well and incubated for 20 min at 37 °C. Next, we rewashed them with serum-free DMEM/F-12 medium and digested cells with trypsin to measure ROS production by flow cytometry (CyFlow® Cube 6, Patec, Nuremberg, Germany). Each exposure group was tested for fluorescence with a density of 1 × 10^4^ cells.

### 2.5. Measurement of Cell Apoptosis Rate

Following 24 h exposure, HCEC grown in the 6-well plate were washed with PBS and harvested then, subsequently, rewashed with precooling PBS, suspended with 1 mL 1× Binding Buffer, then centrifugated them followed by preparing 100 μL cell suspension with the same solution. Next, we added 5 μL Annexin-V FITC and 5 μL PI to the suspension, mixed them thoroughly, and incubated for 15 min at room temperature away from light. Lastly, we supplemented 500 μL PBS to detect fluorescence by flow cytometry. Early apoptotic cells stained by Annexin-FITC showed strong green fluorescence, while late apoptotic cells stained by Annexin-V FITC and PI staining showed double green and red fluorescence. Apoptosis data were analyzed by FlowJo Version 10.4 software (BD Biosciences, Franklin Lakes, NJ, USA).

### 2.6. RNA Extraction, Quantitative Real-Time PCR Analysis

To discover the underlying molecular mechanism, HCEC seeded in a 6-well plate were treated with 1 μM, 10 μM, and 100 μM NiCl_2_ for 24 h after adherence, then we extracted total RNA from them using a total RNA extraction reagent. The content and purity of purified RNA were determined by a NanoPhotometer^®^ N60 (IMPLEN GmbH, Munich, Germany). cDNA was synthesized by reverse transcription using a 20 μL system as required by the Script 1st Strand cDNA Synthesis Kit, and 1000 ng RNA was reversely transcribed into cDNA using the Gradient PCR System (Mastercycler nexus GSX1, Eppendorf, Hamburg, Germany). Apoptosis-related genes were detected by a Roche LightCycler 480II Real-Time PCR system (Roche, Basel, Switzerland) using SYBR green qPCR master mix. The required conditions for determination were as follows: keep 95 °C for 10 min, followed by 40 cycles including 95 °C for 15 s and 60 °C for 1 min. Primer sequences were selected from Harvard PrimerBank (https://pga.mgh.harvard.edu/primerbank, accessed on 1st December 2021). Specific primer sequences are shown in Table 1. The data were calculated by the 2^−ΔΔCT^ method, with the internal reference genes β-actin as the control for normalization of the relative expression level of target genes.

### 2.7. Statistical Analysis

All the experiments were repeated three times. Statistical analysis was assessed using ordinary one-way ANOVA in GraphPad Prism Version 9.1.1 (GraphPad Software, LLC., San Diago, CA, USA). The results are represented as mean with standard deviation, and a *p*-value < 0.05 indicated a significant difference.

## 3. Results and Discussions

### 3.1. Ni exposure Decreased Cell Viability and Altered Cell Morphology

As one of the basic units of the cornea at the front of the ocular surface, human corneal epithelial cells are easily harmed by external pollutants [25], among them, heavy metals can decrease TEER value and impair functions of tight junction and mucin of HCEC [5]. In the same way, further probing the individual effect and toxic mechanism of Ni on HCEC is necessary, which is explicitly conducive to identifying the harm of a single metal on the cornea. As an essential indicator, cell viability can reflect the cytotoxicity of heavy metals effectively; moreover, cell morphology can also be employed as an important index to judge the physiological dysfunction and cytotoxicity of cells [18].

**Figure 1:**
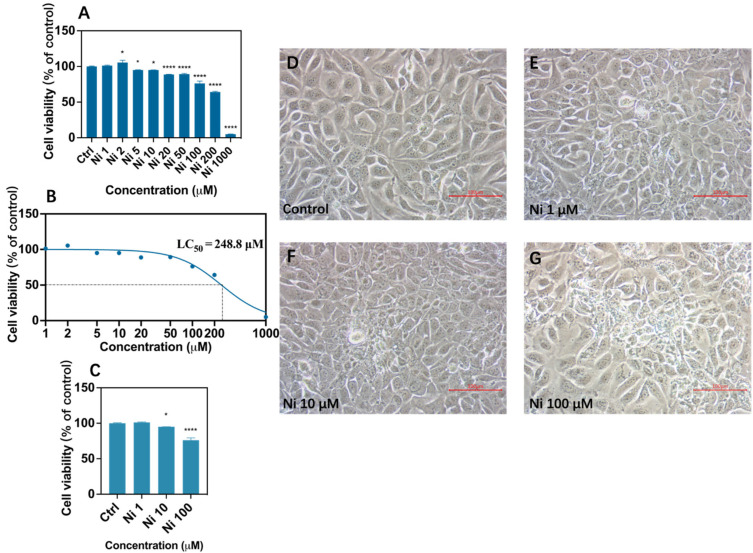
Cell viability after Ni exposure (**A**,**C**) and LC_50_ calculated by nonlinear fitting after logarithmic conversion of concentration after Ni exposure (**B**) and morphological changes of human corneal epithelial cells by inverted microscope (TS-100, Nikon, Tokyo, Japan) at 200× magnification (**D**–**G**) after exposure to Ni for 24 h. Each bar represents the mean ± SD of three replicates. * *p* < 0.05, **** *p* < 0.0001.

This study chose to use corneal epithelial cells isolated from healthy human eyeballs, which are closer to the real physiological function of human beings and can reflect the results more reliably. To decide the final concentrations of Ni to expose, we conducted a preliminary experiment to determine the lethal concentration. It has been verified that in most literature, the exposure duration of HCEC with pollutants of environmental concentration is 24 h [5,18,26], so we chose 24 h as the exposure duration. Additionally, according to the results (Figure 1B), we found out the lethal concentration of 50% (LC_50_) of Ni to HCEC was 248.8 μM; hence, we chose 1 μM (about 1/250 LC_50_), 10 μM (about 10/250 LC_50_), and 100 μM (about 100/250 LC_50_) to explore further. It is proven that released Ni can store up in the human body reaching 20 to 1000 μM [27], so all these concentrations used for further study are all within ambient or in vivo concentrations, and even less than concentrations that can be attained in vivo [28]. Data suggested the LC_50_ of Ni in MCF-7 cells, Hep-2 cells, and HepG2 cells range from 10 to 100 μg/mL [22,29], which revealed that HCEC were relatively sensitive to Ni compared to other cell species. Given that, exploring Ni-induced cytotoxicity of human corneal epithelial cells is needed.

The outcomes implied that there was no significant difference in cell viability between the control group and the 1 μM exposure group (Ni_1_) (Figure 1E) and cells showed no obvious change in cell morphology at 200× magnification. According to former studies, this may be because Ni stimulated the self-defense mechanism in HCEC and maintained the dynamic balance between cells and the external environment [30]. However, the viability of cells exposed to 10 μM Ni (Ni_10_) had declined (Figure 1F), showing a decrease rate of about 5% (Figure 1C). In addition, the minority of cells atrophied into a spindle shape and floated, indicating that Ni at this level made mild corneal toxicity. Ni at 100 μM (Ni_100_) significantly suppressed cell viability to 76% (Figure 1C), and it corresponded to its cellular morphology change, quite a few of the cells displayed an abnormal shape, with rough contour and atrophic form, and some even glowed and floated unusually (Figure 1G). The data showed Ni had certain cytotoxicity at these concentrations and hindered cell proliferation; moreover, its toxicity increased with increasing concentrations. Kim A. et al. (2020) explained that Ni generally enters cells through transferrin or divalent metal transporter 1 (DMT1) and exhibits cytotoxicity by inhibiting DNA repair processes or by inducing oxidative stress through the depletion of intracellular antioxidants such as glutathione [31], but the specifics need to be verified.

### 3.2. Ni Exposure Aggravated Oxidative Damage

Several results showed that cytotoxicity results from Ni exposure are accompanied by oxidative stress. Oxidative stress is an important mechanism for heavy metals to inhibit cell viability [32], while oxidative damage refers to the loss of balance between the level of free radicals and the antioxidant capacity of cells in the process of oxidative stress. Evidence suggests that Ni and its compounds can be dissolved or acidified in cells, then produce superoxide anion free radicals and lipid peroxidation, which are considered to work in disrupting the activities of various antioxidant enzymes in cells, they directly stimulate cells to produce ROS. Eventually, these reactions lead to oxidative damage [22,33].

**Figure 2:**
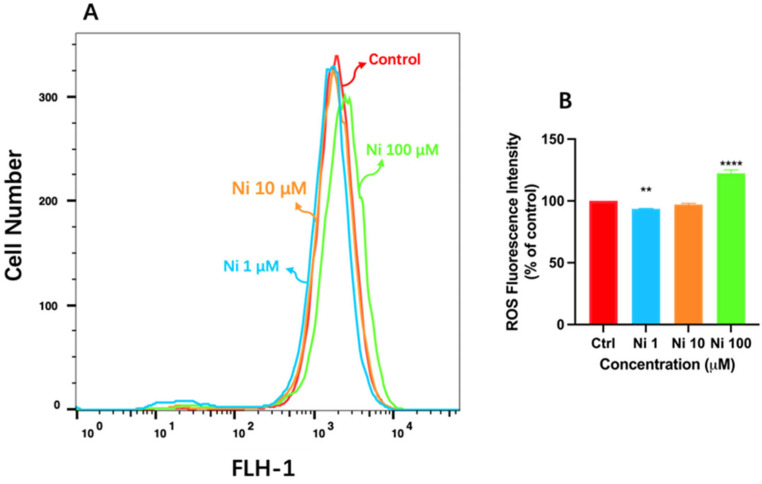
The fluorescence intensity (**A**) detected by flow cytometry via fluorescent probe DCFH-DA of human corneal epithelial cells and its histogram expressed as % of control (**B**) after 24 h exposure. Each bar represents the mean ± SD of three replicates. ** *p* < 0.01, **** *p* < 0.0001.

Thus, to better understand the relationship between cytotoxicity caused by Ni exposure and oxidative damage led by ROS production, we detected intracellular reactive oxygen species by flow cytometry via fluorescent probe DCFH-DA. The peak value of the broken line shifted to the right indicates the increase of ROS fluorescence intensity, so the production of ROS induced by Ni exposure was in a dose-dependent manner (Figure 2); thereinto, the fluorescence intensity of Ni_1_ showed weakened gently compared to the control group, presenting the production of ROS as around 93% (Figure 2B). Likewise, the production rate of ROS in Ni_10_ treatment decreased insignificantly, the value was about 97%. According to available experimental results, this phenomenon may be due to the fact that Ni at low concentrations could impel the intracellular antioxidant mechanism to take effect, prompting the cells to activate the self-defense response, and the antioxidant enzymes produced in this process will combine with Ni^2+^ and remove the ROS generated by oxidative stress [34]. While exposure to Ni_100_ resulted in a significant increase in ROS concentrations up to 122% (Figure 2B), and the fluorescence curve shifted significantly to the right, indicating more ROS were produced under Ni_100_ exposure (Figure 2A), it was consistent with the experimental result reported in the literature that more ROS would be produced as the concentration of Ni increases [35], and excessive production of ROS caused the insufficiency of cell anti-oxidation capacity, which led to oxidative damage.

To sum up, Ni at 100 μM is likely to bring out oxidative damage to HCEC, and this may be attributable to Ni exposure breaking intracellular homeostasis [36]. Remarkably, the generation of ROS is associated with dry eye disease [37], so it is necessary to dig out the underlying relationship between oxidative damage and cell apoptosis caused by Ni in HCEC.

### 3.3. Ni Exposure-Induced Cell Apoptosis

Intracellular reactive oxygen species have been proved by accumulating evidence to perform mediating apoptosis with effect, oxidative stress caused by ROS generation induces mitochondrial perturbation, finally leading to cell apoptosis [38,39]. Ni can produce ROS after entering cells, and then may cause oxidative damage, such as DNA damage, fracture, and base dislocation; eventually, these disorders result in cell apoptosis [40]. In particular, Ni can take effect in mediating abnormal apoptosis in various cell types, and it turned out that ROS at μM levels can lead to cell apoptosis. Cell apoptosis is a programmed cell death that occurs in cellular basic biological processes, playing a critical role in maintaining cellular homeostasis. However, when the regulation of apoptosis is unbalanced, excessive cell proliferation or apoptosis can be caused, resulting in related diseases [41]. Several experiments have been conducted to prove that heavy metals trigger cytotoxicity via giving rise to cell apoptosis [42].

**Figure 3:**
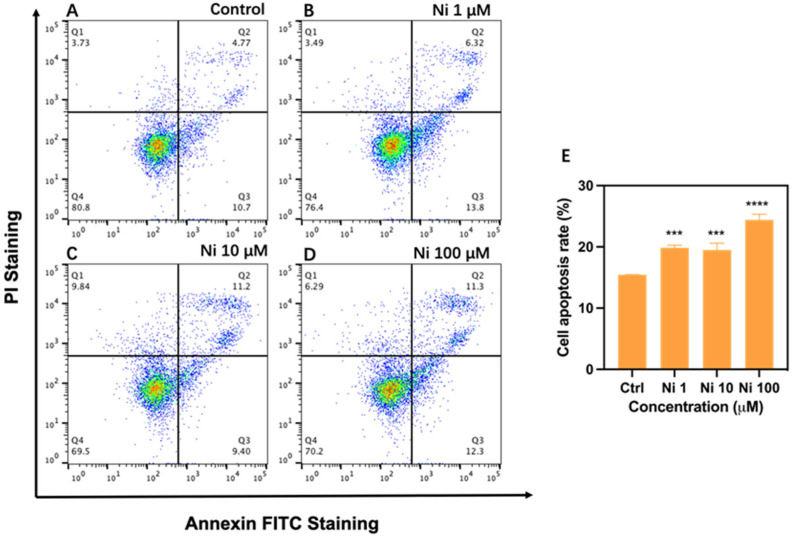
The apoptosis rate of human corneal epithelial cells after exposure to Ni for 24 h measured by flow cytometry (**A**–**D**). And its statistical graph calculated by Q2 + Q3 quadrant (**E**). Each bar represents the mean ± SD of three replicates. *** *p* < 0.01, **** *p* < 0.0001.

In the present study, we utilized Annexin-V FITC/PI staining to discover the apoptosis rate of Ni-exposed HCEC by flow cytometry. In summary, the apoptosis ratios of different exposed groups showed the same upward trend (*p* < 0.001) and a dose-independent increase (Figure 3). Both Ni_1_ and Ni_10_ activated cell apoptosis of HCEC, and the apoptosis ratios of both were all elevated to around 19%. Though these two groups did not generate more ROS, there may exist other factors inducing apoptosis, whether they caused apoptosis through the intrinsic mitochondria-dependent pathway is needed to be proven in the molecular aspect. Ni_100_ enhanced the number of apoptotic cells with the ratio increasing to 24% (*p* < 0.001), and both FITV-positive and PI-positive cells were promoted in this group, hinting at more grievous injury in HCEC. Corresponding to that, ROS level also presented a clear rise, implying these two things were interrelated. It turns out that there is a similar case, Ni at achievable concentrations in vivo has been proved to induce neutrophils apoptosis by ROS overexpression [28]. According to extant research, we infer that excessive ROS produced by Ni_100_ results in apoptotic cell death [43], but its related molecular mechanism deserves further study.

Taken together, Ni exposure is related to cell apoptosis induction in HCEC, the higher the concentration, the more severe the apoptosis, in addition to this, Ni-induced apoptosis in HCEC is relevant to oxidative damage. 

### 3.4. Ni Exposure Up-Regulated mRNA Expression of Apoptosis

To be better acquainted with the underlying molecular mechanism of apoptosis induced by Ni, we determined mRNA expression of apoptosis-related genes (*Caspase-8*, *Caspase-9*, *NF-κB*, *IL-1β*, and *Caspase-3*) in HCEC following 24 h exposure. As expected, all five genes were up-regulated in Ni-treated groups in a concentration-dependent manner; however, the up-regulation was most significant in the Ni_100_ group.

**Figure 4:**
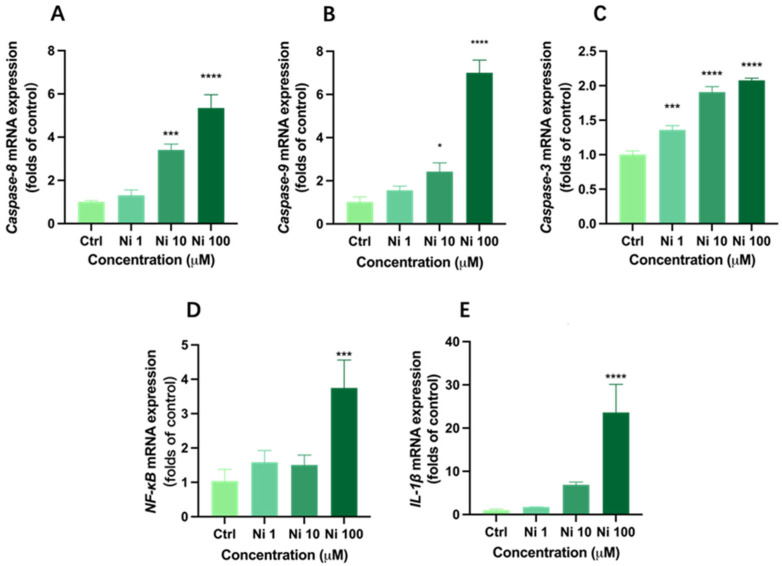
Expression of apoptotic genes *Caspase-8* (**A**), *Caspase-9* (**B**) and *Caspase-3* (**C**), and inflammatory genes *NF-κB* (**D**) and *IL-1β* (**E**) in human corneal epithelial cells after exposure to Ni for 24 h. Each bar represents the mean ± SD of three replicates. * *p* < 0.05, *** *p* < 0.001, **** *p* < 0.0001.

*Caspase-8* and *Caspase-9* are both apoptosis promoters. They recognize and activate other downstream Caspase-family genes through protein-protein interaction, such as *Caspase-3* [44]. In general, *Caspase-8* is closely related to apoptosis of the death receptor pathway, while *Caspase-9* is involved in apoptosis through the mitochondrial pathway. What is more, *Caspase-3* plays a key role in the execution of cell apoptosis and can simultaneously mediate the apoptosis signal of the mitochondrial pathway and death receptor pathway; moreover, its expression level is positively correlated with the apoptosis rate [45]. Based on our experimental data, there were no significant increases in expression levels of *Caspase-8* and *Caspase-9* under Ni exposure at 1 μM, while *Caspase-3* mRNA expression presented an upward trend of 1.36 times (*p* < 0.05). As for the Ni_10_ group, *Caspase-8*, *Caspase-9*, and *Caspase-3* were relatively significantly expressed, and the Ni_100_ group showed a relatively high expression of *Caspase-8* by 5.34 times (Figure 4A), and the mRNA expressions of *Caspase-9* and *Caspase-3* in the Ni_100_ group were 7.01 and 2.08 times, respectively (Figure 4B,C), indicating the occurrence of cell apoptosis. Additionally, the expression levels of these apoptosis-related genes were positively correlated with Ni concentration.

*Caspase-8* is a major factor not only in death receptor signal transduction but also in *NF-κB* nuclear translocation and activation [46]. *NF-κB* plays an important pro-inflammatory role in inducing many diseases and is related to the expression of pro-inflammatory cytokine *IL-1β* in epithelial cells [47]. In addition, it can be regulated by oxidative modification. Zheng et al. (2018) have proved that the overexpression of *IL-1β* poses a high risk for corneal health, which may cause dry eye [48]. Corresponding to *Caspase-8* expression, the expression of *NF-κB* increased by 3.73 times and *IL-1β* expression significantly increased by 23.63 times after Ni_100_ exposure (*p* < 0.0001) (Figure 4D,E). *IL-1β* does not follow the traditional secretion pathway, its secretion is determined by the intensity of extracellular stimulation [49], sustained activation of *NF-κB* can result in its high expression level [50], and our results are consistent with this statement. Furthermore, up-regulation of *IL-1β* is associated with *Caspase-8*-induced apoptosis [51].

Collectively, it can be inferred that Ni at high concentrations can increase *NF-κB* through up-regulating *Caspase-8* gene expression, and then promote the expression of *IL-1β* and *Caspase-3*, leading to apoptosis through the death receptor pathway [52]. At the same time, the up-regulation of *Caspase-9* may be due to the consistency of the time and precise location of expression of *Caspase-8* and *Caspase-9*; furthermore, *Caspase-8* can indirectly act in the intracellular apoptosis pathway under certain conditions, leading to the activation of *Caspase-9* [53]. Once activated, *Caspase-9* also cleaves and stimulates the downstream effector protein *Caspase-3*, thereby causing endogenous apoptosis. While the expression levels of *NF-κB* and *IL-1β* in Ni_1_ and Ni_10_ groups were not significantly up-regulated, *Caspase-8*, *Caspase-9*, and *Caspase-3* were relatively distinctly expressed. Combined with previously measured apoptosis rates, it is sufficient to prove that Ni could likewise induce apoptosis at low concentrations. Considering that up-regulation of *Caspase-8* can induce apoptosis through a variety of pathways, we conclude that Ni can both lead to apoptosis through the intrinsic mitochondrial receptor pathway and extrinsic death receptor pathway, thereinto, relatively high concentrations of Ni may represent a potential hazard to the cornea through triggering dry eye, but the specific mechanism needs to be further studied.

## 4. Conclusions

In this study, Ni decreased the cell viability in a concentration-dependent manner, disturbed the balance of intracellular oxidation and antioxidant system, and aggravated oxidative damage, ultimately leading to cell apoptosis through the pathway activated by ROS, carrying a risk of dry eye. Given that, more attention should be paid to corneal injury caused by Ni pollution in the environment. Nevertheless, the specific mechanism of Ni damage to the cornea and the deeper molecular mechanism of corneal apoptosis caused by Ni still needs to be further addressed.

## Figures and Tables

**Table 1:** The primers for RT-qPCR.

	Forward Primer	Reverse Primer
β-actin	GACATCCGCAAAGACCTG	GGAAGGTGGACAGCGAG
NF-κB	GAAGAAAATGGTGGAGTCTG	GGTTCACTAGTTTCCAAGTC
IL-1β	AGCTACGAATCTCCGACCAC	CGTTATCCCATGTGTCGAAGAA
Caspase-3	CATGGAAGCGAATCAATGGACT	CTGTACCAGACCGAGATGTCA
Caspase-8 Caspase-9	CGGACTCTCCAAGAGAACAGG	TCAAAGGTCGTGGTCAAAGCC
	CTCAGACCAGAGATTCGCAAAC	GCATTTCCCCTCAAACTCTCAA

## Data Availability

Data are contained within the article.
